# Cardiologists' Perspectives on Race-Based Drug Labels and Prescribing Within the Context of Treating Heart Failure

**DOI:** 10.1089/heq.2018.0074

**Published:** 2019-05-22

**Authors:** Shawneequa L. Callier, Brooke A. Cunningham, Jill Powell, Mary Anne McDonald, Charmaine D.M. Royal

**Affiliations:** ^1^ Department of Clinical Research and Leadership, George Washington University School of Medicine and Health Sciences, Washington, District of Columbia.; ^2^ Center for Research on Genomics and Global Health, National Human Genome Research Institute, National Institutes of Health, Bethesda, Maryland.; ^3^ Department of Family Medicine and Community Health, University of Minnesota Medical School, Minneapolis, Minnesota.; ^4^ Center on Genomics, Race, Identity, Difference, Duke University, Durham, North Carolina.; ^5^ Department of African & African American Studies, Duke University, Durham, North Carolina.

**Keywords:** race, health disparities, BiDil, heart-failure

## Abstract

**Purpose:** Cardiologists are known to consider patients' race when treating heart failure, but their views on the benefits and harms of this practice are largely undocumented. We set out to explore cardiologists' perspectives on the benefits and harms of race-based drug labels and guidelines. Specifically, we focused on isosorbide dinitrate and hydralazine hydrochloride (sold in a patented form as BiDil), a combination of drugs recommended for the treatment of black patients receiving optimal medical therapy for symptomatic heart failure and reduced ejection fraction.

**Methods:** We conducted 81 semistructured interviews at an American College of Cardiology Annual meeting to assess cardiologists' and cardiology fellows' attitudes toward the use of race in drug prescribing. Investigators reviewed and coded the interviews using inductive qualitative analysis techniques.

**Results:** Many participants believed that race-based drug labels might help doctors prescribe effective medications to patients sooner. More than half of the participants expressed concerns, however, that considering race within the context of treating heart failure could potentially harm patients as well. Harms identified included the likelihood that patients who could benefit from a drug may not receive it because of their race; insufficient understanding about gene–drug–environment interactions; and simplistic applications of race in the clinic.

**Conclusions:** Few participants expressed approval of using race in drug prescribing without recognizing the potential harms, yet most participants stated that they continue to consider race when prescribing isosorbide dinitrate and hydralazine hydrochloride. Within the context of treating heart failure, more open discussions about the benefits and harms of race-based drug labels and prescribing are needed to address cardiologists' concerns.

## Introduction

Debate on the medical relevance of race is well documented^1–3^ and ongoing.^4,5^ Due to advancements in genomic technology and the alarming discovery that genomic research overwhelmingly focuses on populations of primarily European ancestry, the issue of the use of race in medicine has received increased attention in just the past year.^4^ Studies report diverging views on the role of race in drug prescribing among family practitioners and internists, many of whom are on the frontlines of identifying patients with heart failure. Primary care physicians and cardiologists are typically tasked with prescribing isosorbide dinitrate and hydralazine hydrochloride as part of patients' heart failure regimens. Absent from the literature is an analysis of cardiologists' perspectives on the role of race-based guidance that focuses on black patients. There is a need to provide more empirical evidence to inform new policies and guidelines around how to approach race and ethnicity in research and medicine.

As specialists well versed in the intricacies of treating heart failure, cardiologists' views can inform debate and policy resolution in this area. This study aimed to qualitatively assess cardiologists' beliefs about the benefits and harms of race-based drug labels and indications. As evidenced by the United States Census, racial categories belie complex social and scientific interactions and change in meaning over time.^6^ Yet, the use of race in drug prescribing persists.^7^ Within the context of treating hypertension, physicians continue to report that African American patients are less responsive than white patients to angiotensin converting enzyme inhibitors, leading to the concern that patients who identify as black or African American and might benefit from these drugs will not receive them.^8^

We focused on isosorbide dinitrate and hydralazine hydrochloride, which is FDA approved and recommended by expert panels for self-identified black patients on optimal medical therapy for symptomatic heart failure and reduced ejection fraction.^8–11^ In 2005, the FDA approved the first race-based drug, BiDil, a combination of two generics, isosorbide dinitrate and hydralazine hydrochloride, for self-identified black patients.^9,10^ As others have explained in detail elsewhere, the African American Heart Failure Trial (A-HeFT), which led to the approval of BiDil, was launched to demonstrate the effectiveness of an isosorbide dinitrate and hydralazine hydrochloride combined pill in self-identified black patients with heart failure, and the investigators deemed the trial a success.^11^ While the first Vasodilator Heart Failure Trial (V-HeFT I) confirmed the benefit of treating patients with mild-to-severe heart failure with a combined dosage of isosorbide dinitrate and hydralazine hydrochloride, the second trial, V-HeFT II, showed that enalapril provided more of a mortality benefit than isosorbide dinitrate and hydralazine hydrochloride within a general patient population. In an apparent effort to patent and market the combination pill, the makers of BiDil eventually conducted a retrospective analysis of the V-HeFT trials breaking down the results by race.^12^ In the ensuing A-HeFT trial, investigators reported a 43% relative decrease in the mortality rate among black patients. The A-HeFT trial, however, did not investigate whether black patients benefited more than other groups from BiDil or the underlying reasons for the mortality reduction.^11,13^ FDA argued that these limitations did not justify precluding BiDil's approval for black patients only,^10^ but others have questioned the appropriateness of the use of race as a proxy for alternative, well-defined contributors to drug response.^3^

Among internists, perspectives on the benefits and harms of race-based drug labels and prescribing are complex. Maglo et al. found, for instance, that internists surveyed prescribed and were willing to prescribe BiDil more often to black patients than to white patients,^14^ yet, many of those surveyed were either unaware of the controversy around BiDil or undeterred by it and prescribed BiDil along racial lines. In a study focused on race-based drug prescribing in general, Bonham et al. found that black and white internists believed that race is relevant to drug prescribing,^7^ yet, the investigators also observed an unwillingness among respondents to purport a connection between race, genetics, and disease. Still, Bonham et al. reported in a national survey of general internists that most respondents were comfortable with collecting patients' race and ethnicity. Respondents who were more comfortable collecting such data or who did so directly were more likely to use race in clinical decision-making than respondents who were uncomfortable.^15^ Other studies show that family practitioners and primary care doctors also have complicated ideas about the role of race in drug prescribing. When reporting on views about race and ethnicity, Warshauer-Baker et al. reported that family practitioners generally believed that both are as relevant to differences in health outcomes as genetic and environmental influences, but few of them rated race or ethnicity as essential to clinical care.^16^ Meanwhile, Frank et al. reported that physicians were wary that race reflected meaningful genetic differences,^17^ felt concerned that BiDil's approval related to commercial interests, and believed that medication effectiveness varied across racial groups.

## Methods

### Design

Our objective was to examine cardiologists' and trainees (i.e., cardiology fellows) perspectives on race-based drug labels and prescribing within the context of treating patients with heart failure and what they thought about race-based drugs in the multiracial population of the United States. Gaining access to physicians for interviews or surveys is difficult, and response rates are typically very low, under 30%.^18,19^ We wanted to learn the cardiologists' experiences using the drug BiDil and their attitudes and beliefs about race-based drug indications. Because we wanted to learn about attitudes and beliefs in the cardiologists' own words, we believed that interviewing physicians face-to-face would be more effective than using a survey.^20,21^

We contacted the American College of Cardiology (ACC) and received permission to conduct brief in-person interviews at their annual meeting held in Washington, DC from March 29 to 31 in 2014. Participants provided verbal consent at the start of the interview and the interviews were recorded. The Duke University Institutional Review Board approved the study.

### Sample and recruitment

Interviewers used convenience sampling to recruit attendees at the ACC conference during conference hours in the exhibit hall which was accessible only by registered conference attendees. This was the only area in which we were permitted to conduct interviews. If a prospective participant agreed to be interviewed, we determined eligibility by asking each person if he or she was a cardiologist or cardiology fellow practicing in the United States (see screening questions in Table 1). Importantly, the interviewers decided to wait for the interviewee to bring up the subject of race so as to not bias or interfere with the interviewee's natural response on this topic. The interviewers asked specific questions about race at the end of the interview if they were not spontaneously answered during the conversation. The majority of the attendees were white men, and so, we attempted to oversample people of color and women (Table 2). All three interviewers agreed to oversample by prioritizing interviews with cardiologists who were women and/or persons of color. In terms of sample size, we decided to conduct as many interviews as possible since our time to collect data was limited to the two and a half days of the conference.

**Table 1.** American College of Cardiology Interview Guide, March 29–31, 2014

Screening questions
1. Are you a cardiologist?
2. Do you practice in the United States?
3. Did you ever prescribe BiDil or the generics?
Interview questions
4. What was your primary factor in choosing who should receive it?
5. Do you prescribe it only to black patients?
6. Any opinions about it only being approved for blacks?
7. What were your colleagues saying about it?
8. Do you see any benefits/harms to having race-specific drugs?
9. What are your thoughts about expanding the BiDil indication for all races?
10. Are you using BiDil? On all patients or just black patients?
11. Do you use patient's race to determine treatment?
a. How do you determine their race?

Primary questions included in semistructured interview guide. Interviewers probed for more information when time permitted or shortened the interview if the participant had time constraints. Demographic questions related to practice area, age, gender, self-identified race, and years of practice.

**Table 2.** Participant Demographics (*N*=81)

		*n* (%)
Race/Ethnicity	Asian	14 (17)
	Black or African American	15 (19)
	Hispanic/Latino	3 (4)
	White	44 (54)
	Middle Eastern	4 (5)
	Other	1 (1)
	Missing	1 (1)
Age	35 or younger	21 (26)
	36–45	18 (22)
	46–65	35 (43)
	Over 65	5 (6)
	Missing	2 (2)
Gender	Male	73 (90)
	Female	8 (10)
Practice environment (some cardiologists practice in more than one type of environment)	Hospital	17 (21)
	Group/private practice/clinic	48 (60)
	Academic	31 (38)
	Community	30 (37)
	Government	1 (1)
	Missing	11 (14)
Years of practice	1–5	30 (37)
	6–10	5 (6)
	11–20	9 (11)
	20+	31 (38)
	Missing	6 (7)

### Data collection

Three investigators (Callier, Cunningham, and McDonald) conducted in-person interviews at the conference using established qualitative research methods. We used a semistructured interview guide with three screening questions and eight interview questions developed by the interviewers with the PI (Royal). Each interviewer individually approached a conference participant, identified herself as a researcher, asked if she could conduct a short (5–10 min) interview, told the participant that responses would be anonymous (we would not ask their names or where they worked) and asked permission to record the interview with compact digital recorders. We then asked cardiologists questions, such as, whether they prescribe BiDil or isosorbide dinitrate and hydralazine hydrochloride, what they thought about race-based drug labels and BiDil, and whether race-based drug labels are beneficial or harmful (see the interview guide in Table 1). In addition, we inquired about the factors that influence decision-making about whether to prescribe BiDil and/or its generic components to patients, including the roles of medical conditions and race. The length of the interviews varied based on the availability of the interviewee to discuss questions in depth and their interest in the topic.^20^

Qualitative research is often used to “facilitate study of issues in depth and detail”^20^ rather than to collect data to extrapolate to a population. A semistructured, interview guide lists questions or topics of interest, but, unlike surveys, the questions can be asked in whatever order is appropriate for each interviewee, and it is not necessary to use the same wording. Interviewing with a semistructured guide is flexible and fluid and resembles a conversation; it is a framework for gathering data, not a rigid list of questions.^20^ At the end of each interview, we collected demographic information (e.g., self-identified race/ethnicity, age, practice setting, and type). Interviews lasted from 5 to 15 min and were later transcribed verbatim. As an incentive, interviewees could enter a raffle for the chance to win an Apple iPad. We collected identifiers for those who wanted to enroll in the raffle, but these identifiers were not linked to transcripts.

The interview transcripts were reviewed and coded, using *NVivo 10.0*, by three team members (M.A.M., S.L.C., J.P.), with one (M.A.M.) acting as a lead coder. We used the interview guide to develop initial codes, identify emergent codes as coding proceeded, and apply the codes as needed. We defined each code and referred to these definitions as we applied the codes so that their meaning remained stable. All data coded by secondary coders were reviewed by the primary coder to ensure consistent coding of the entire data set.^20,22–25^ Coded data were extracted to an excel table and then sorted and compared by the lead author (S.L.C.).

To further ensure internal consistency of qualitative data analysis, we performed interrater reliability (IRR) tests on five full interview transcripts, as well as on the assignment of a subset of key codes from 11 additional transcripts. Scores from IRR tests, which ranged from 50% to 100%, with a majority of agreement scores falling between 87% and 100%, indicated strong agreement (>70%) among coders. After we coded about a quarter of the material, we reviewed data at each code to ensure consistency with code definitions. Data were moved to more appropriate codes when necessary or removed from the coded material. When we completed the coding, we again reviewed data at each code as described above to ensure that they fit within the code definition.^25^

## Results

We interviewed a total of 81 cardiologists from a variety of racial and ethnic backgrounds, age groups, and practice settings, but the overwhelming majority of participants were white men (Table 2). Participants included fellows as well as established specialists. As a whole, responses varied within demographic groups, and there were no significant between-group differences.

Participants expressed approval and disapproval of race-based drug labels in degrees. Responses ranged from full approval of race-specific drug indications to approval with reservations among those who acknowledged the drawbacks to complete disapproval. Notably, nearly half of all participants expressed skepticism or strongly disapproved of race-based drug labels and the use of race in drug prescribing. Thus, there was no overwhelming majority among respondents who felt either way. The following themes emerged from the interviews that capture the reasons for participants' views.

### Benefits of race-based drug prescribing

Our screening questions specifically asked whether potential participants had prescribed BiDil. BiDil was used as an example, for instance, when discussing the benefits of race-based drug labels and their impact on prescribing. Those who favored BiDil's race-based indication usually cited data published about the A-HeFT clinical trial as support for their views. Such respondents believed that the A-HeFT clinical trial provided evidence that BiDil provides a mortality benefit to black patients with heart failure. Some of these participants contended that most clinical trials involve white, male, patient groups, and that the A-Heft study presented an important opportunity to address the needs of black patients who, they emphasized, suffer immense health disparities in health outcomes. Capturing this sentiment, one cardiologist explained:

“A lot of the studies, at least the older studies, really haven't addressed the race issues in terms of differences in morbidity and mortality in terms of the disease state, so I think that we have a lot to learn about it. This was one of the earlier studies that seemed to indicate that there was an advantage in blacks so I don't have a problem with it.”

Other participants believed that race-based drug prescribing has played a role in reducing the extent of trial and error drug prescribing. In the following quote, a cardiologist refers to BiDil as an “ethnic drug” (equating race with ethnicity) and states that this designation is justified in its labeling if the right treatment is prescribed to black patients sooner:

Well, I think the ethnic drug in specifically the African American population has been useful… it may make you more apt to try the drug earlier rather than later in the course of an algorithm for controlling hypertension or treating [Chronic Heart Failure] CHF.

In a similar vein, some participants weighed the potential to successfully treat heart failure with ejection fraction in black patients as more important than any concerns that they may have about the use of race. As one cardiologist asserted, race-based drug prescribing could be justified by positive outcomes.

“At the end of the day, my job as a physician is to help people live their lives better, keep them healthy, have them live longer if that's what they want. And if this [race-based drug prescribing] works for patients that are black, then for my patients that come see me, it's something that I certainly should consider as a physician.”

A subset of participants argued that isosorbide dinitrate and hydralazine hydrochloride may work better or sooner for black patients, but the reasons they believed that this was true varied. One cardiologist explained, for instance, “there are physiologic differences as well as all the other socioeconomic things and if it works, it works,” when providing perspective on FDA approval of a drug for patients of one particular race.

When asked about whether the label for BiDil should be expanded to include other or all racial groups, many participants asserted that any expansion of the label for BiDil should be evidence-based.

Interviewer: [I]f an Asian person comes in with significant heart failure, are you going to think about BiDil with that person?

Respondent: Well, hopefully, not. Because I think we are [under] so much pressure to do guideline-based, and also evidence-based, and some people would say that the evidence for using in other races is not so strong.

Cardiologists noted that they may prescribe off-label to nonblack patients. Others were concerned that insurance companies may refuse to cover prescriptions for nonblack populations.

### Disadvantages of race-based drug prescribing

More than half of our participants either voiced some concern about race-based drug labels or described them as completely inappropriate. The most common concern articulated was that doctors may fail to prescribe isosorbide dinitrate and hydralazine hydrochloride to other nonindicated populations who may benefit. Providing such a view, the cardiologist quoted below explained that BiDil, in particular, could provide benefits to patients who are not black, but it may not be prescribed for them.

“…we see it time and time again with BiDil. Even though it's labeled to benefit African Americans more, Caucasian patients do benefit from it.”

A different cardiologist pointed to the design of the A-HeFT trial, which only included African Americans as the source of the limitation, and argued that race is too simple a category.

“I think it is too simplistic to do race-based therapy… whoever made that trial, made it in such a way that they were not giving us an opportunity to select more specifically who should and shouldn't get it”

Other cardiologists noted that a variety of biological and concomitant medical factors contribute to heart failure morbidity and mortality, and a more rigorous trial would have taken other factors into account to better inform prescribing.

Numerous participants felt that cardiologists risk making an inaccurate determination about the best course of treatment for a patient based on the “blanket designation” of race. Participants provided a number of reasons as to why this is problematic. As one cardiologist stated, “[i]t's one's experience and social position, for instance, rather than genes that can cause a person to identify as African American.” Another participant reflected on the fact that a patient may be “20 percent African and 80 percent Irish.” Along the same vein, a cardiologist expressed the belief that there is not “just one racial type and that's the problem. There's a lot of mixing.” Suggesting that both black and nonblack patients are at risk, one cardiologist argued, BiDil may not be the best option for a particular black patient, but the provider may prescribe it anyway based on race.

“Well, I think the problem is that it sort of has gotten pigeon holed into, here's what's good for African Americans, here's what's good for white people, to the point that maybe you are excluding the other therapies, which probably should not be done.”

In a few cases, participants acknowledged the widespread mixed continental and genetic ancestry of patients living in America. Such participants often expressed the view that there is still more to learn about the differences in drug response among patients of diverse ancestry, including black and white patients. Cardiologists expressed different opinions, however, about the role of race in drug response, with some arguing more confidently than others that race tells us little about patient biology. Some noted that BiDil is one of few options for advanced heart failure, regardless of race.

Finally, some cardiologists feared that race-based drug labels could agitate patients or cause anxiety among providers. A patient may feel, for instance, that he or she is not getting the best possible medical care because of the provider's focus on race. As one cardiologist explained, such patients could feel isolated. Meanwhile, the physician may feel uncomfortable discussing race with patients.

## Discussion

As a whole, we gained some useful insight into perspectives on race-based drug labels and how race influences cardiologists' drug prescribing practices, but there were some limitations to the study. Study participation was limited to a convenience sample of cardiologists attending the national ACC meeting. Therefore, our results may not be generalizable to cardiologists who are not engaged in the discourse on race and drug-prescribing or those who do not typically attend such conferences. In addition, we were unable to obtain full and complete answers from all interviewees or recruit a substantial number of cardiologists who were women and/or racial and ethnic minorities.

Still, our interviews of cardiologists attending the 2014 ACC conference identified two major quandaries related to race-based drug labels. First, participants both advocated for pharmaceutical studies that consider race and warned that race-based research results should be critically evaluated. Cardiologists in our study pointed to the A-HeFT clinical trial results as justification for prescribing isosorbide dinitrate and hydralazine hydrochloride mostly or only to black patients. Furthermore, they appeared to be so compelled by the published literature on isosorbide dinitrate and hydralazine hydrochloride that they advocated for maintaining the race-based indication until efficacy in other racial groups could be proved. At the same time, as other authors have found with respect to internists' perspectives,^15^ they expressed concern about the potential harmful consequences of complying with the drug label. Specifically, some participants believed that all patients risk insufficient or incorrect treatment due to race-guided approaches to drug prescribing.

Uncertainty about the role of race in drug response has contributed to limited options for clinicians who believe that racial groups are not biologically distinct but also believe that prescribing based on race is better than not considering race at all. Unfortunately, clinical research to definitively assess the consequences of this particular practice is nonexistent. Equally concerning is that a race-based drug label has been used in the case of BiDil to establish patent protection on combinations of generic drugs to extend its profitability.^10^ Many participants in this study clearly articulated the need for a new paradigm that goes beyond race. This view is consistent with recent advocacy by scholars of diverse disciplines: academics and clinicians have made an urgent plea to the scientific community to reexamine the use of race in biomedical research and clinical care.^4,5^ This plea has reverberated in the peer-reviewed literature for decades and has reemerged recently in response to an increasingly pervasive and powerful era of genomic testing that has failed to include populations of diverse ancestry in research.^4^ There is widespread agreement that diversity and inclusion in biomedical research is required to help reduce health disparities, but the path forward with regard to when and how to examine the role of race in research and medicine remains obscure.^4,5^

Due to the “one-drop” rule in the United States, the racial category “African American” does not represent individuals' diverse ancestry, which often includes ancestry from European and other populations. Furthermore, researchers and clinicians may use the terms “black” and “African American” interchangeably and may not consider the social and cultural aspects of this distinction. At the same time, African Americans experience disturbing health disparities that scientists do not fully understand.^1^

New research is prioritizing broader characteristics about individuals to advance precision medicine, a medical approach that seeks to improve diagnosis, prevention, and therapy.^26^ Recent scholarship has highlighted the pressing need for the scientific community to revisit, examine, and clarify the role of race in research and medicine given these new opportunities in precision medicine research.^27^ Improved personalized management of heart failure, for instance, would involve a large spectrum of potential applications inclusive of genomic, environmental, and social and behavioral considerations.^26–30^ If the role of race in medical care is mischaracterized, however, precision medicine may fall far short of its goals.

Developments in precision medicine could cause further challenges for cardiologists if pharmaceutical companies continue to use race on patent applications, promoting the notion that race is biologically or pharmacogenomically relevant.^2,31,32^ While BiDil remains the only FDA-approved drug indicated for one race, there are other drugs that are touted as more effective in particular racial groups, such as the prescription eye drop, Travatan.^33^ Even without exclusively approving medications by race, many drug companies report racial differences in pharmacokinetics, safety, and efficacy on drug labels.^32^ For example, AstraZeneca sought to obtain approval for a race-based indication for a drug for Asian patients, but FDA rejected the proposal.^33^ As biomedical researchers emphasize the importance of multivariate influences on drug response and health outcomes, comfort with race-based prescribing may diminish within a medical system that is increasingly emphasizing integrative molecular and environmental algorithms for profiling patients. Since studies have shown how pharmacogenomic alleles can differ in frequency among ethnic groups that are traditionally lumped under one race (i.e., the Maasai versus the Luhya ethnic groups' 13.6% vs. 3.3% frequency of the HLA-B*5701 pharmacogenomic allele in Kenya),^34^ continued reliance on race could slow progress and lead to medical errors.

As our results show, physicians sometimes engage in race-based drug prescribing in practice for a number of reasons. If it is true that physicians generally aspire to provide targeted therapies based on high-quality evidence, then advancements in cardiovascular medicine, precision medicine, and genomics should minimize the role of race in drug prescribing. The time is ripe to reexamine guidelines that emphasize racial differences in drug prescribing and consider integrated approaches that target the root causes of health disparities.

## Abbreviations Used

A-HeFT: African American Heart Failure Trial
ACC: American College of Cardiology
IRR: interrater reliability
V-HeFT: vasodilator heart failure trial
